# Tumor-targeted IL-12 combined with tumor resection yields a survival-favorable immune profile

**DOI:** 10.1186/s40425-019-0631-z

**Published:** 2019-06-17

**Authors:** Qingnan Zhao, Jiemiao Hu, Abhisek Mitra, Jeffry Cutrera, Wendong Zhang, Zhongting Zhang, Jun Yan, Xueqing Xia, Kris Michael Mahadeo, John Andrew Livingston, Richard Gorlick, Shulin Li

**Affiliations:** 1 Department of Pediatrics–Research, The University of Texas MD Anderson Cancer Center, Houston, TX 77030 USA; 2 Pharmaceutical company of Pfizer in Pearl River, New York, NY 10965 USA; 3 Department of Gynecologic Oncology and Reproductive Medicine, The University of Texas MD Anderson Cancer Center, Houston, TX 77030 USA; 4 Department of Pediatric Stem Cell Transplantation and Cellular Therapy, The University of Texas MD Anderson Cancer Center, Houston, TX 77030 USA; 5 Department of Sarcoma Medical Oncology, The University of Texas MD Anderson Cancer Center, Houston, TX 77030 USA; 6 Department of Pediatrics–Research, The University of Texas MD Anderson Cancer Center, 1515 Holcombe Boulevard, Unit 853, Houston, TX 77054 USA

**Keywords:** Tumor-targeted IL-12, Surgical resection, Immune profile, Overall survival

## Abstract

**Background:**

Although accumulated evidence provides a strong scientific premise for using immune profiles to predict survival in patients with cancer, a universal immune profile across tumor types is still lacking, and how to achieve a survival-associated immune profile remains to be evaluated.

**Methods:**

We analyzed datasets from The Cancer Genome Atlas to identify an immune profile associated with prolonged overall survival in multiple tumor types and tested the efficacy of tumor cell-surface vimentin–targeted interleukin 12 (ttIL-12) in inducing that immune profile and prolonging survival in both mouse and patient-derived xenograft tumor models.

**Results:**

We identified an immune profile (IFNγ^Hi^CD8^Hi^FOXP3^Low^CD33^Low^) associated with prolonged overall survival across several human tumor types. ttIL-12 in combination with surgical resection of the primary tumor transformed tumors to this immune profile. Intriguingly, this immune profile transformation led to inhibition of metastasis and to prolonged survival in both mouse and patient-derived xenograft malignant models. Wild-type IL-12 combined with surgery was significantly less effective. In the IL-12–sensitive C3H mouse strain, in fact, wild-type IL-12 and surgery resulted in shorter overall survival than in mice treated with control pDNA; this surprising result is believed to be attributable to IL-12 toxicity, which was absent in the mice treated with ttIL-12. The ttIL-12–induced immune profile associated with longer overall survival was also associated with a greater accumulation of CD8^+^ T cells and reduced infiltration of regulatory T cells, myeloid-derived suppressor cells, and tumor-associated macrophages. The underlying mechanism for this transformation by ttIL-12 treatment was induction of expression of CXCL9 and reduction of expression of CXCL2 and CCL22 in tumors.

**Conclusions:**

ttIL-12 when combined with surgery led to conversion to the IFNγ^Hi^CD8^Hi^FOXP3^Low^CD33^Low^ immune profile, eliminated relapse and metastasis, and prolonged overall survival.

**Electronic supplementary material:**

The online version of this article (10.1186/s40425-019-0631-z) contains supplementary material, which is available to authorized users.

## Introduction

Communication between tumor cells and the associated immune system is pivotal for tumor metastasis, relapse, and progression. Since Rudolf Virchow first proposed the link between tumorigenesis and chronic inflammation, myriad studies have contributed to the characterization of immune cell–associated tumor progression and treatment resistance [1–3]. Some of these studies have shown that a favorable immune environment may boost a tumor’s response to treatment [4], including chemotherapy [5], immunotherapy [6, 7], and irradiation [8]. These observations reinforce the need to better understand the prognostic impact of immune cells on the tumor and its microenvironment and on clinical outcome.

The primary immune cells in tumors are tumor-infiltrating lymphocytes, myeloid cells, and macrophages, which consist of both immunostimulatory populations and immunosuppressive populations. Accumulating evidence shows that immunostimulatory populations, such as CD8^+^ T cells, natural killer (NK) cells, type 1 helper T cells, and M1 macrophages, efficiently boost antitumor immune responses [9]. Immunosuppressive populations, including myeloid-derived suppressor cells (MDSCs), regulatory T cells (Tregs), and tumor-associated macrophages, are mobilized during tumorigenesis and relapse. After infiltrating into developing tumors, MDSCs promote tumor vascularization and disrupt immunosurveillance, including M1 macrophage polarization, antigen presentation [10], T cell activation [11], and NK cell cytotoxicity [12]. Similar to MDSCs, Tregs suppress tumor-associated antigen presentation and disrupt cytotoxic T cell function [13]. Tregs regulate the expansion and activation of T and B cells and have a pivotal role in maintaining the homeostasis of innate cytotoxic lymphocytes [14]. In various reports, a high frequency of CD8^+^ tumor-infiltrating lymphocytes [15] or a low frequency of MDSCs [16] or FOXP3^+^ Tregs [17] in tumors was associated with longer survival in the targeted tumor types. However, the optimal immune profile for effectively predicting patient survival across tumor types and the approaches to achieving such an optimal immune profile are largely unknown.

Interleukin 12 (IL-12), which bridges adaptive and innate immune responses, is accepted as a central antitumor immunotherapeutic agent. Although systemic delivery of IL-12 (e.g., IL-12–secreting T cells) has substantial anticancer efficacy, it causes significant toxic effects in humans [18]. To ameliorate this toxicity, we developed an IL-12 that targets cell-surface vimentin (CSV), a protein found on the surfaces of tumor cells across tumor types, especially metastatic tumors [19, 20]. Administration of tumor CSV-targeted IL-12 (ttIL-12), a fusion gene that encodes IL-12 and a comprehensive CSV-targeting carcinoma homing peptide, increased tumor accumulation of IL-12 in vivo [19], and ttIL-12 therapy via intramuscular electroporation promoted the inhibition of tumor growth with less liver toxicity than wild-type IL-12 (wtIL-12) therapy [19]. While ttIL-12 may modify dendritic cells in tumors [19], its overall impact on the tumor immune profile has not been investigated.

Our purpose in this study was to identify an immune profile that is consistently associated with longer survival across different cancers and to investigate a therapeutic intervention that can transform a short overall survival (OS) immune profile into the long OS immune profile. By analyzing The Cancer Genome Atlas (TCGA) data across different human tumors for markers of immunostimulatory interferon-gamma (IFNγ)–positive CD8^+^ cytotoxic T cells and immunosuppressive Tregs and MDSCs, we found that the immune profile IFNγ^Hi^CD8a^Hi^FOXP3^Low^CD33^Low^ predicted excellent patient outcome across different tumor types. Notably, ttIL-12 converted immune profiles associated with shorter survival to the immune profile associated with longer survival in two independent mouse tumor models (one mesenchymal tumor and one epithelial tumor) and one patient-derived xenograft (PDX) model (osteosarcoma). Mechanistic studies found that ttIL-12 treatment increased the expression of T cell–recruiting chemokine CXCL9 and IFNγ and reduced the expression of MDSC- and Treg-recruiting CXC-chemokine ligand 2 (CXCL2) and CC-chemokine ligand 22 (CCL22) in tumors. Overexpression of CXCL2 or CCL22, or depletion of CD8a, impaired ttIL-12’s efficacy in suppressing tumor growth and metastasis, which validated the novel mechanism of ttIL-12 in converting a short OS immune profile to a long OS immune profile.

## Materials and methods

### TCGA data mining

Gene expression and survival data were obtained from the TCGA portal (http://www.cbioportal.org/public-portal/). Copy numbers of the CD8a, IFNγ, CD33, and FOXP3 genes and survival data were obtained for eight cancer types: breast adenocarcinoma, sarcoma, lung adenocarcinoma, liver cancer, head and neck squamous cell carcinoma, colon carcinoma, ovarian carcinoma, and melanoma.

### Plasmid DNA preparation

All gene constructs were generated as previously described [19]. The wild-type IL-12 plasmid DNA (pDNA), CCL22 DNA (pCCL22), and CXCL2 DNA (pCXCL2) were obtained from Genscript (New Jersey, USA), and the ttIL-12 pDNA was described previously [19]. The control pDNA (Ctrl) was wtIL-12 with the IL-12 gene deleted.

### Cell lines

The 4 T1 murine breast cancer cell line and LM8 murine osteosarcoma cell line were obtained from ATCC (Manassas, VA). Both cell lines were maintained in Dulbecco modified Eagle medium containing 10% fetal bovine serum (Life Technologies, Carlsbad, CA) at 37 °C and in an atmosphere containing 5% CO_2_.

### Animals and tumor models

BALB/c and C3H female mice were obtained from The Jackson Laboratory (Bar Harbor, ME), and CB17SC *scid*^*−/−*^ female mice were purchased from Taconic Farms (Germantown, NY). All were 6 to 8 weeks old upon initiation of the experiments. Detailed information can be found in the supplementary material.

In brief, orthotopic 4 T1 and LM8 tumors were initiated by inoculating 1 × 10^5^ cells in the third mammary fat pads of the BALB/c mice and in the right tibia of C3H mice, respectively. The first pDNA treatments (10 μg; wtIL-12, ttIL-12, or Ctrl) via intramuscular electroporation were performed as described previously [19]; a second identical treatment was administered 10 days later. For metastatic tumor analysis, 4 T1-bearing mice and LM8-bearing mice were euthanized 20 days or 5 days after primary tumor removal, respectively, and lungs, livers, and bones were collected to analyze metastatic status. India ink inflation was performed to determine the level of lung metastasis, and white metastatic nodules were counted using a dissecting microscope.

To overexpress CCL22 and CXCL2 in vivo, 10 μg pCCL22 or pCXCL2 was administered via intramuscular electroporation into 4 T1- and LM8-bearing mice 3 days prior to ttIL-12 treatment as described above. Seven days after the second treatment, orthotopic 4 T1 and LM8 tumors were collected for later analysis.

For the PDX model, patient-derived OS60-SJ osteosarcoma tumor cells (generously provided by Dr. Richard Gorlick, the Pediatric Preclinical Testing Consortium, The University of Texas MD Anderson Cancer Center) were implanted subcutaneously into CB17SC *scid*^*−/−*^ female mice. When tumors reached 300 mm^3^ in size, the mice were treated with Ctrl, human wtIL-12, or human ttIL-12 pDNA as already described once per week for 4 weeks. To make these CB17SC *scid*^*−/−*^ mice immunocompetent, they were injected with 2 × 10^7^ human peripheral blood mononuclear cells (PBMCs) intravenously every 2 weeks along with pDNA treatment.

### Immunohistology staining

Formalin-fixed, paraffin-embedded lung and liver tissue sections were stained with hematoxylin and eosin (H&E; Sigma Chemical, St Louis, MO). Frozen tumor sections were fixed with cold acetone, acetone plus chloroform (1:1), and acetone. Tissue sections were blocked with blocking buffer (5% normal horse serum and 1% normal goat serum in phosphate-buffered saline solution) and incubated with rat anti-mouse CD8α antibody (clone YTS105.18; AbD Serotec, Raleigh, NC). For FOXP3 staining, sections were blocked with blocking buffer containing 0.1% Triton X-100 for 40 min and incubated with rat anti-FOXP3 antibody (#12653; Cell Signaling Technology, Danvers, MA) overnight at 4 °C. The next day, tissue sections were blocked and incubated with goat anti-rat horseradish peroxidase secondary antibody (Life Technologies) for 1 h at room temperature, which was followed by DAB staining for 5~10 min at room temperature. Nuclei were then counterstained with hematoxylin (Sigma Chemical), and tumor sections were mounted with ClearMount Mounting Solution (Life Technologies). Slides were visualized under a Nikon eclipse Ti fluorescence microscope (Nikon Instruments, Melville, NY).

### Immune cell depletion in vivo

During the mouse model experiment just described, an antibody depleting CD8^+^ T cells (250 μg per mouse; clone 2.43) was administered intraperitoneally to mice twice a week for 3 weeks starting on day 3 after tumor cell inoculation.

### Flow cytometry

Portions of primary tumor and metastatic tissues were digested with collagenase IV/DNase (0.2 mg/mL and 10 μg/mL) at 37 °C for 30 min. Cells were put through 40-μm filters to obtain single-cell suspensions and stained as previously described [21]. Cell surfaces were stained with anti-mouse CD4, anti-mouse CD206, anti-mouse CD11b, anti-mouse F4/80, anti-mouse Gr1.1, anti-mouse CD3, anti-mouse CD45, anti-mouse CD8a, anti-mouse CD28, anti-mouse NKG2D, anti-human CD45, anti-human CD8, anti-human CD33, and anti-human HLA-DR antibodies (BioLegend, San Diego, CA) labeled with fluorescein isothiocyanate, PerCP/Cy5.5, phycoerythrin, or Brilliant Violet 421. Flow cytometry was performed using an Attune flow cytometer (Life Technologies), and the data were analyzed with FlowJo software.

### RNA isolation and quantitative real-time PCR

Total RNA was isolated from tumor tissues using TRIzol reagent (Thermo Fisher Scientific, Waltham, MA) according to the manufacturer’s instructions. Equal amounts of RNA were reverse-transcribed into cDNA by using iScript™ gDNA Clear cDNA Synthesis Kit (Bio-Rad, Berkeley, CA). qRT-PCR was performed using the Power SYBR Green Master kit (Thermo Fisher Scientific, Waltham, MA), with a StepOnePlus™ Real-Time PCR System (Thermo Fisher Scientific, Waltham, MA), as previously described [22]. The level of mRNA expression was normalized to that of the housekeeping gene GAPDH. The primer sequences of all mouse genes can be found in supplementary materials.

### Enzyme-linked immunosorbent assay

The IL-12 and IFNγ proteins in mouse tumors were measured in triplicate by commercially available kits (R&D Systems, Minneapolis, MN) by following the manufacturer’s instructions. Mouse CCL22, CXCL9, CXCL10, CCL5, and CXCL2 proteins were assayed by using commercial kits from Biolegend (mouse proinflammatory chemokine mix and match subpanel).

### Statistical analysis

For each reported result, data were pooled from at least two independent experiments. The Student *t*-test was used to compare results between two treatment groups; one-way ANOVA was used to compare results from more than two treatment groups. The statistical significance of differences in survival curves was determined by log-rank survival analysis. All quantified data are presented as mean ± standard deviation (SD) or as indicated. The Prism software (GraphPad Software, Inc., La Jolla, CA) was used to determine the *P* values, and P values < 0.05 were considered statistically significant.

## Results

### An immune profile associated with longer overall survival

Mutation-induced oncogene activation and tumor suppressor gene inactivation have been widely investigated as markers to predict survival duration for patients with cancer. However, no one mutation is known to universally predict OS duration across different tumor types. This is not surprising, because different tumors may utilize different pathways or oncogenes to drive tumor progression and metastasis. However, immune profile infiltration is a common factor across all tumor types. Indeed, some studies have found that a certain type of immune profile was associated with long-term OS [23, 24]. T cell infiltration and frequency of FOXP3^+^ Tregs, MDSCs, and tumor-associated macrophages all have been discussed in the literature as predictors of OS in specific tumor types [25, 26], but none were analyzed across multiple types of tumors. Our first goal for this study was to identify a single immune profile that can universally predict long-term OS across different tumor types. Our second goal was to develop a simple immune therapy that converts tumors from an immune profile associated with short OS to the immune profile associated with longer OS.

**Fig. 1:**
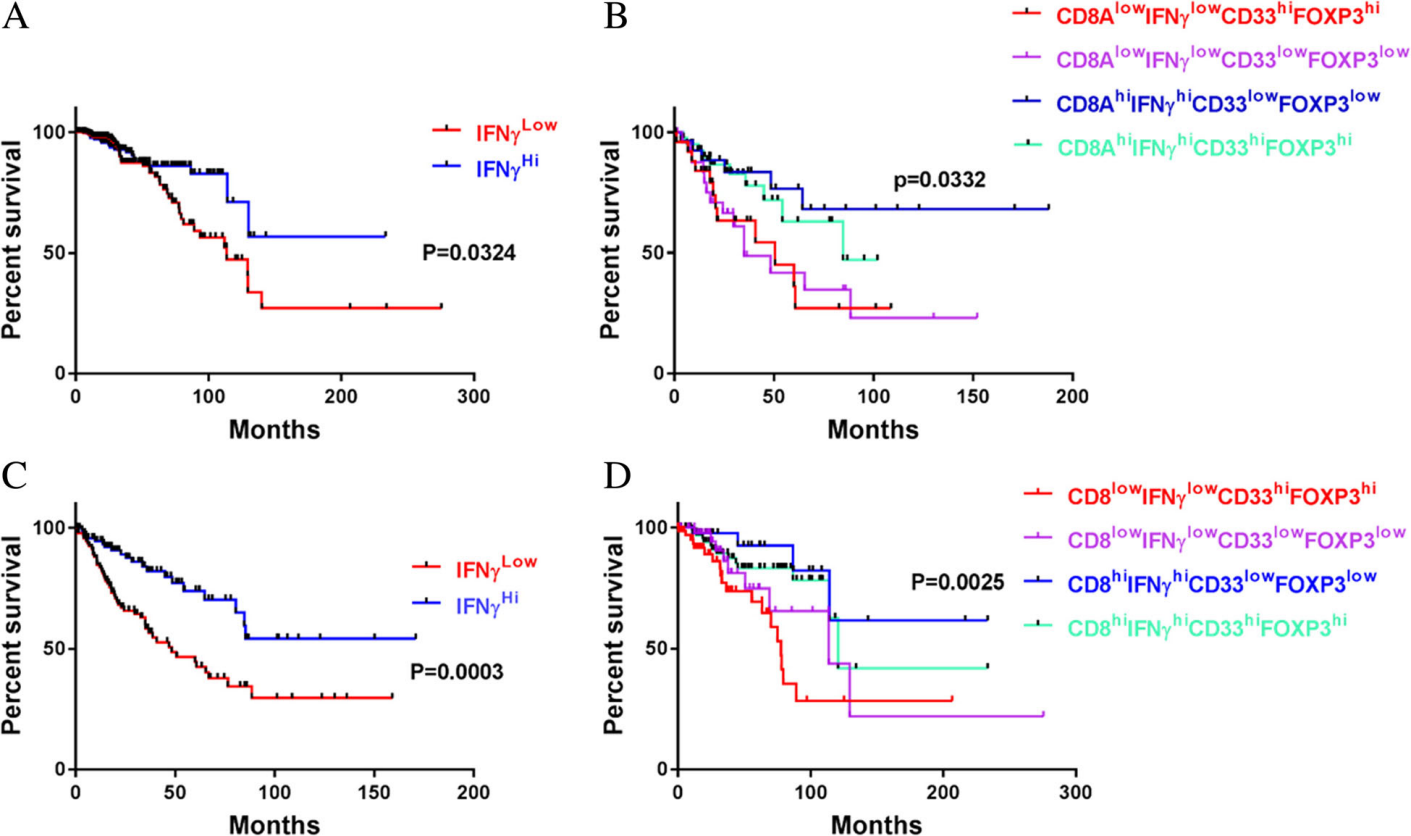
The CD8^Hi^IFNγ^Hi^CD33^Low^FOXP3^Low^ immune profile is correlated with favorable clinical outcome. **a,c** Kaplan-Meier survival analysis of human sarcoma (**a**) and breast carcinoma (**c**) based on IFNγ gene copy number. The overall survival rates were stratified by IFNγ gene expression level: the 25% of patients with the highest IFNγ levels were placed in the IFNγ^Hi^ group and the 25% with the lowest IFNγ levels were placed in the IFNγ^Low^ group. **b,d** Patients with sarcoma (**b**) and patients with breast cancer **d** were grouped by IFNγ, CD8, CD33, and FOXP3 gene expression levels: the 25% of patients in the IFNγ^Hi^ group with the highest CD8a levels were placed in the CD8^Hi^IFNγ^Hi^ group, and the 25% of patients in the IFNγ^Low^ group with the lowest CD8a levels were placed in the CD8^Low^IFNγ^Low^ group. The CD8^Hi^IFNγ^Hi^ group and the CD8^Low^IFNγ^Low^ group were then substratified into the CD8^Hi^IFNγ^Hi^CD33^Low^FOXP3^Low^ group, the CD8^Hi^IFNγ^Hi^CD33^Hi^FOXP3^Hi^ group, the CD8^Low^IFNγ^Low^CD33^Low^FOXP3^Low^ group, and the CD8^Low^IFNγ^Low^CD33^Hi^FOXP3^Hi^ group. Kaplan-Meier analyses were used to determine the association of each group with a favorable survival outcome

To identify an immune profile associated with prolonged survival, we focused on T cell activation markers and immunosuppressive cell markers in an analysis of TCGA data. Our rationale was that functional T cells in tumors are effective in eliminating tumor cells and enhancing responses to chemotherapy, radiation, or other treatments. Although CD69, CD29, and CD25 are T cell activation markers, these markers are found in many other cells [27–29]. IFNγ is also not T cell specific, but its expression in T cells and other immune cells is a fairly good indicator of survival. In fact, IFNγ^Hi^ alone is reasonably accurate in predicting OS rate (Fig. 1a,c), but the predicted OS rates are not very high in some types of tumors (Additional file 1: Table S1). For example, the OS rates of patients with IFNγ^Hi^ lung cancer or melanoma were 23.6.4 and 39.7%, respectively (Additional file 1: Table S1). To refine the profile toward longer OS, we added the CD8 gene; the combination of IFNγ^Hi^ and CD8^Hi^ improved accuracy of identification of patients with longer OS, but further inclusion of parameters indicating low expression levels of immunosuppressive cell markers, such as FOXP3 and CD33, even more greatly improved the accuracy of the combination’s prediction of longer OS. For example, including these two immunosuppressive markers in the profile increased the predicted OS rate from 54.1 to 68% in patients with sarcoma (Fig. 1b,d). This observation makes sense because CD33 is a key marker for MDSCs and FOXP3 is a key indicator for Tregs, and both Tregs and MDSCs can shut down T cell activity by secreting unfavorable molecules such as IL-10, transforming growth factor beta (TGF-β), IL-35, and arginase 1 (Arg1) [30, 31]. Of course, inclusion of additional genes may further improve the accuracy of prediction of OS rate, but further expansion of the combination of biomarkers would have hampered utility for OS analysis across tumor types in the TCGA database, because some datasets lack data on RNAs or genes of interest. Expanding the biomarker panel may also limit its clinical application. However, these four genes, at the indicated expression levels, together predict long-term OS in multiple types of tumors (Additional file 1: Table S1). Our results strongly indicate that the CD8a^Hi^IFNγ^Hi^CD33^Low^FOXP3^Low^ immune profile is associated with long-term OS across multiple tumor types.

### ttIL-12 treatment converts primary tumors to the immune profile associated with long-term OS

The consistent association of the CD8a^Hi^IFNγ^Hi^CD33^Low^FOXP3^Low^ immune profile with long-term OS suggests that any treatment that can convert tumors to this immune profile may prolong OS. In our attempts to identify such a treatment, we have investigated several reagents, but all failed. This study describes our investigation of the impact of ttIL-12 on the immune profiles of two different common tumors. ttIL-12 (US patent 9,657,077 B2) was selected because this product targets a universal tumor-specific protein, CSV, and promotes accumulation of IL-12 in tumors [19].

We first assayed the effect of ttIL-12 on tumor progression using two aggressive orthotopic mouse tumor models: an epithelial tumor (4 T1, breast carcinoma) and a mesenchymal tumor (LM8, sarcoma). BALB/c mice bearing a 4 T1 tumor and C3H mice bearing an LM8 tumor were treated with empty (Ctrl), wtIL-12, or ttIL-12 pDNA. As expected, ttIL-12 treatment showed higher potency in suppressing tumor growth than wtIL-12 treatment in both 4 T1 and LM8 models (Additional file 1: Figure S1).

**Fig. 2:**
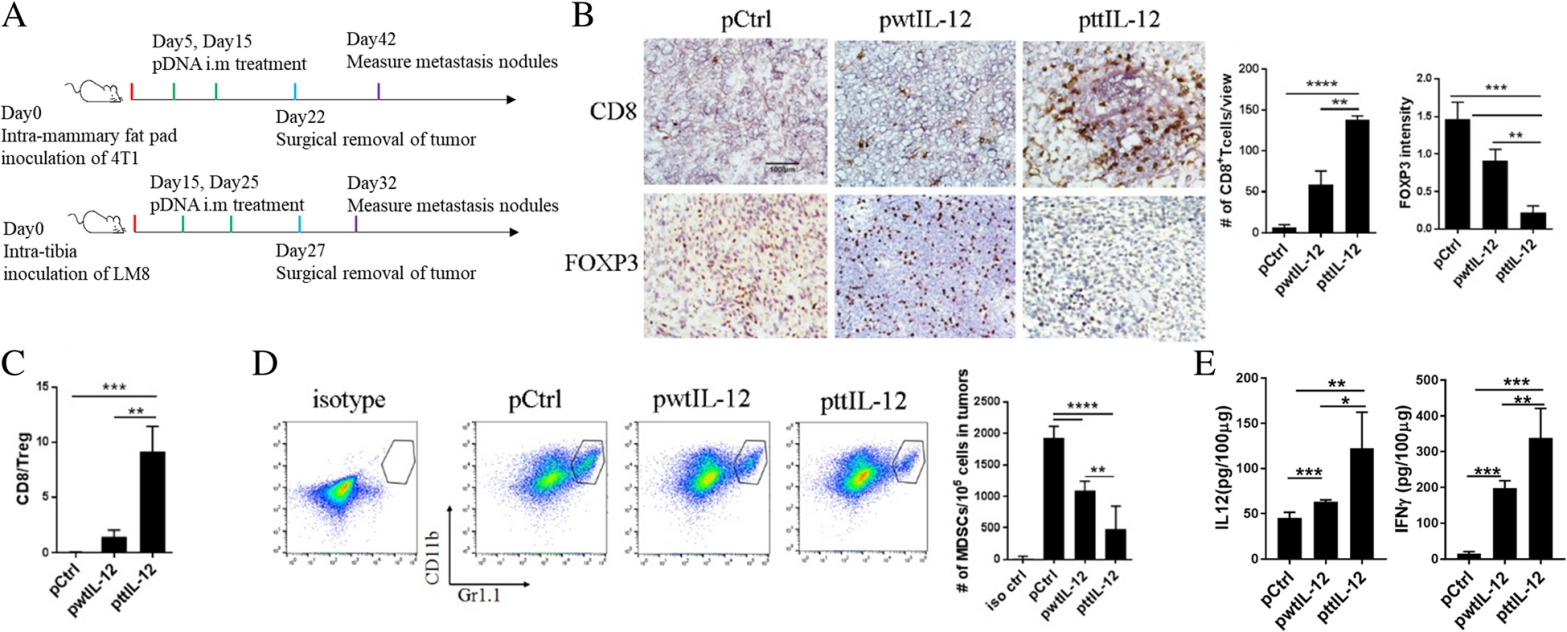
ttIL-12 enhanced infiltration of CD8^+^ T cells into tumors, decreased infiltration of Tregs and MDSCs, and increased tumor levels of IFNγ. (**a**) Treatment scheme. BALB/c mice were inoculated with 4 T1 breast cancer cells by intra-mammary fat pad injection. CH3 mice were inoculated with LM8 sarcoma cells by intratibial injection. Mice from both groups were treated with empty control plasmid DNA (pDNA; pCtrl), wild-type IL-12 pDNA (pwtIL-12), or tumor-targeted IL-12 pDNA (pttIL-12; *n* = 5~8 mice per treatment group). Primary tumors were removed surgically as indicated. **b** Frozen sections from 4 T1 primary tumors were stained with CD8 antibody (upper panels) or FOXP3 antibody (lower panels). FOXP3 is a marker of T regulatory cells (Tregs). Representative sections are shown. Scale bars, 100 μm. Absolute numbers of CD8^+^ cells and FOXP3^+^ cells were determined by microscopy. **c** Calculated CD8^+^/Treg ratios. **d** Representative flow cytometry plots of percentages of CD11b^+^Gr1^+^ cells (myeloid-derived suppressor cells [MDSCs]); absolute numbers of MDSCs per 10^5^ total tumor cells were calculated from the flow cytometry data. **e** Levels of the IL-12 and IFNγ proteins in primary 4 T1 tumors were measured by ELISA. *P < 0.05, **P < 0.01, ***P < 0.001, ****P < 0.0001

We then evaluated the effect of ttIL-12 on immune profile in tumors. CD8^+^ T cells, Tregs, and MDSCs were assayed using antibodies against CD8a, FOXP3, and CD11b/Gr1, respectively. To our surprise, ttIL-12 treatment not only increased numbers of CD8^+^ T cells but also decreased numbers of FOXP3^+^ Tregs and MDSCs in 4 T1 tumors compared with both Ctrl treatment and wtIL-12 treatment (Fig. 2). ttIL-12 treatment also decreased M2 macrophages in tumors, as determined by using antibodies against F4/80/CD11b/CD206 (Additional file 1: Figure S2). Similar results were obtained in the LM8 model (Additional file 1: Figure S3). These results suggest that ttIL-12 has extra independent biological function on top of the known IL-12 function, which is to block immune suppression. We also measured the tumor levels of IL-12 and IFNγ proteins, which can overcome the suppressive tumor microenvironment and shift the balance to favor CD8^+^ T cell antitumor immunity. Our data show that tumors treated with ttIL-12 accumulated more IL-12 and expressed higher levels of IFNγ than those treated with wtIL-12 (Fig. 2e; *P* = 0.0024). Notably, the ratio of CD8^+^ T cells to Tregs in ttIL-12–treated tumors was significantly higher than in wtIL-12–treated tumors (Fig. 2c; *P* = 0.0092), which shows that ttIL-12 has higher potency than wtIL-12 therapy in inducing the immune profile associated with longer OS.

To expand our evaluation of ttIL-12 treatment, the antitumor effect of wtIL-12 and ttIL-12 on human tumor progression was measured in an osteosarcoma PDX model. According to our data, the antitumor effect of ttIL-12 is dependent on the immune system. Therefore, human PBMCs were injected in combination with the pDNA into CB17SC *scid*^*−/−*^ mice bearing an OS60-SJ osteosarcoma tumor. The tumors treated with ttIL-12 showed dramatically higher levels of IFNγ than those treated with Ctrl (*P* < 0.05), higher frequency of CD8^+^ T cells (*P* = 0.0358), and lower frequencies of MDSCs (*P* = 0.0458) and Tregs (*P* = 0.0059) (Additional file 1: Figure S8), suggesting that ttIL-12 treatment converted the immune profile in these osteosarcoma PDX tumors to the IFNγ^Hi^CD8^Hi^FOXP3^Low^CD33^Low^ immune profile associated with longer OS.

### ttIL-12 treatment followed by surgery suppresses metastasis in two independent tumor models

**Fig. 3:**
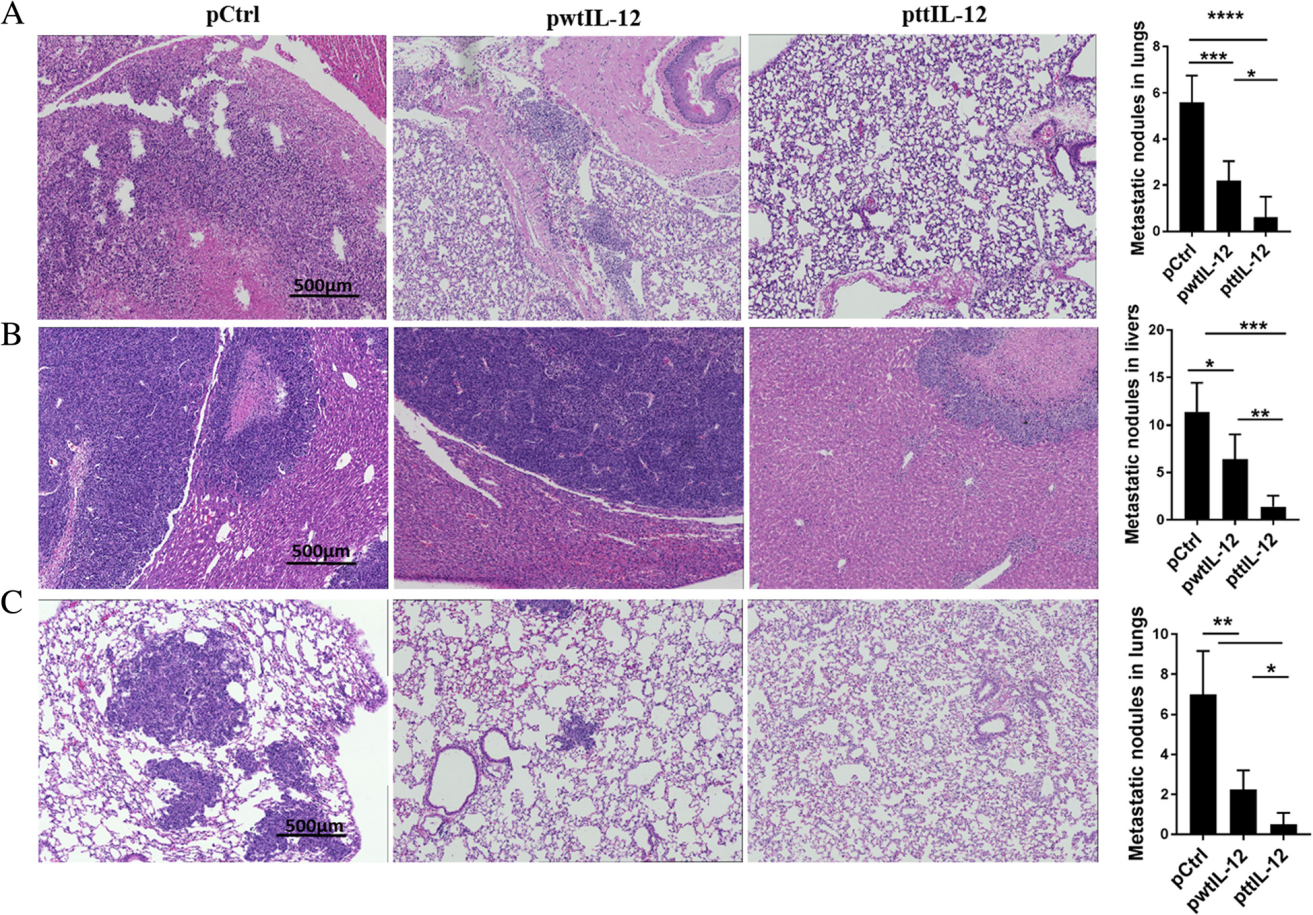
ttIL-12 induced a greater anti-metastatic effect than wtIL-12. Tumor-bearing mice were treated with control pDNA (pCtrl), wild-type IL-12 pDNA (pwtIL-12), or tumor-targeted IL-12 pDNA (pttIL-12). **a** Lungs were removed from 4 T1 tumor–bearing mice and were sectioned and stained with hematoxylin and eosin (H&E) to detect metastatic nodules. Representative sections are shown. Scale bars, 500 μm. **b** Livers and (**c**) lungs were removed from LM8 tumor–bearing mice, sectioned, and stained with H&E to detect metastatic nodules. Representative sections are shown. Scale bars, 500 μm for H&E and low-magnification immunohistochemistry. *P < 0.05, **P < 0.01, ***P < 0.001, ****P < 0.0001

To investigate the potential impact of the combination of ttIL-12 treatment and surgery on metastasis, 4 T1 or LM8 tumor–bearing mice treated with ttIL-12, wtIL-12, or Ctrl underwent surgical removal of the primary orthotopic tumor after the pDNA treatment, and the metastatic tumor nodules in their lungs and livers were counted 20 days (4 T1) or 5 days (LM8) later. Numbers of metastatic nodules in the lungs were lower in the 4 T1 mice treated with ttIL-12 plus surgery (ttIL-12 + S) than in those treated with wtIL-12 plus surgery (wtIL-12 + S), as validated via H&E staining in lungs and livers. Numbers of lung metastatic nodules were dramatically lower in 4 T1-bearing mice treated with ttIL-12 + S than in those treated with control plasmid plus surgery (Ctrl+S), while wtIL-12 + S slightly decreased lung metastatic nodules in both 4 T1 and LM8 tumor–bearing mice (Fig. 3a, c). Moreover, ttIL-12 + S decreased liver metastatic nodules in the LM8 model compared with wtIL-12 + S (Fig. 3b; *P* = 0.0044). Together, these data show that ttIL-12 + S had greater anti-metastatic effect than wtIL-12 + S in both sarcoma and breast carcinoma models.

### ttIL-12 treatment converts metastatic tumors from short- to long-term OS immune profile

**Fig. 4:**
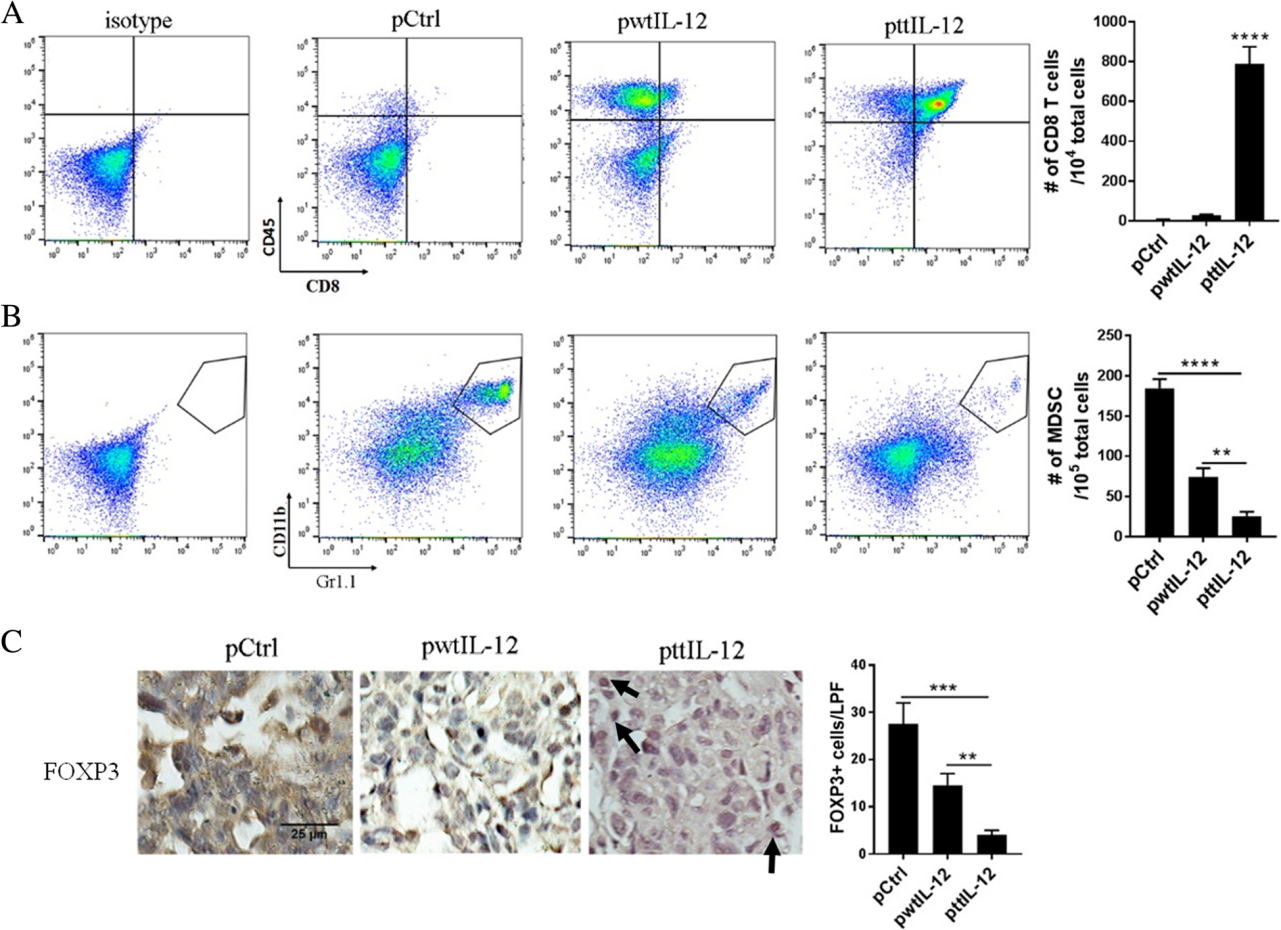
ttIL-12 increased CD8^+^ T cell infiltration and decreased MDSC and Treg infiltration into lung metastatic nodules. **a,b** Mice bearing LM8 tumors were treated with empty plasmid DNA (pCtrl), wild-type IL-12 plasmid DNA (pwtIL-12), or tumor-targeted IL-12 plasmid DNA (pttIL-12). At the end of the treatment period, their lungs were removed and subjected to flow cytometry to determine percentages of (**a**) CD8^+^CD45^+^ cells (CD8^+^ T cells) and (**b**) CD11b^+^Gr1^+^ cells (myeloid-derived suppressor cells [MDSCs]) in metastatic nodules, and absolute numbers of CD8^+^ T cells and MDSCs were calculated from the flow cytometry data. **c** Sections of paraffin-embedded lungs from LM8 tumor–bearing mice were labeled with FOXP3, and absolute numbers of FOXP3^+^ cells in metastases were determined by microscopy. Representative images are shown. Scale bars, 25 μm. Pooled data from two independent experiments with *n* = 3 mice per treatment group. Means ± standard error of the mean are shown. **P < 0.01, ***P < 0.001, ****P < 0.0001

To identify the mechanism that may account for the enhanced anti-metastatic effect of ttIL-12 + S, we analyzed the metastatic nodules in lungs and livers of LM8 tumor–bearing mice for infiltrated CD8^+^ T cells, MDSCs, and Tregs using flow cytometry and immunohistochemical staining. Mice treated with ttIL-12 + S showed higher frequencies of CD8^+^ T cells (*P* < 0.0001) and lower frequencies of MDSCs (*P* < 0.0001) and Tregs (*P* < 0.001) in lung metastatic nodules than mice treated with wtIL-12 + S (Fig. 4, Additional file 1: Figure S4), which was consistent with ttIL-12’s role in inducing the CD8a^Hi^IFNγ^Hi^CD33^Low^FOXP3^Low^ immune profile in primary orthotopic tumors. Similar results were found in the liver metastatic nodules of LM8 tumor–bearing mice (Additional file 1: Figure S5).

Previous reports showed that infiltrated NKG2D^+^CD8^+^ T cells in tumors exert effector functions in the tumor microenvironment, attack tumor cells, induce tumor cell death, and significantly halt tumor progression [22]. To understand whether these infiltrated CD8^+^ T cells in metastatic nodules were tumor-responsive or exhausted, we analyzed the metastatic nodules for CD28 and NKG2D, co-stimulatory receptors that serve as non-exhaustion markers. 4 T1 tumor–bearing mice treated with ttIL-12 + S showed higher proportions of both CD28^+^CD8^+^ T cells and NKG2D^+^CD8^+^ T cells in lung metastatic nodules than mice treated with wtIL-12 + S or Ctrl+S (Additional file 1: Figure S4). Similarly, ttIL-12 + S increased numbers of NKG2D^+^CD8^+^ T cells in livers of LM8 tumor–bearing mice (Additional file 1: Figure S5A), showing that ttIL-12 + S induced its greater anti-metastatic effect, compared with wtIL-12 + S, through activating non-exhausting CD8^+^ T cells.

To further validate the significance of CD8^+^ T cells in the anti-metastatic effect of ttIL-12, both 4 T1 and LM8 tumor–bearing mice were treated with CD8-depleting antibody along with ttIL-12. As expected, blocking CD8 significantly dampened the survival-favorable effect of ttIL-12 treatment in both models (Fig. 6a, b; *P* < 0.0001 and *P* = 0.0105, respectively), suggesting that CD8^+^ T cells are pivotal in the suppression of tumor progression by ttIL-12 + S.

### ttIL-12 treatment increases chemokines that recruit CD8^+^ T cells but reduces chemokines that recruit MDSCs and Tregs

**Fig. 5:**
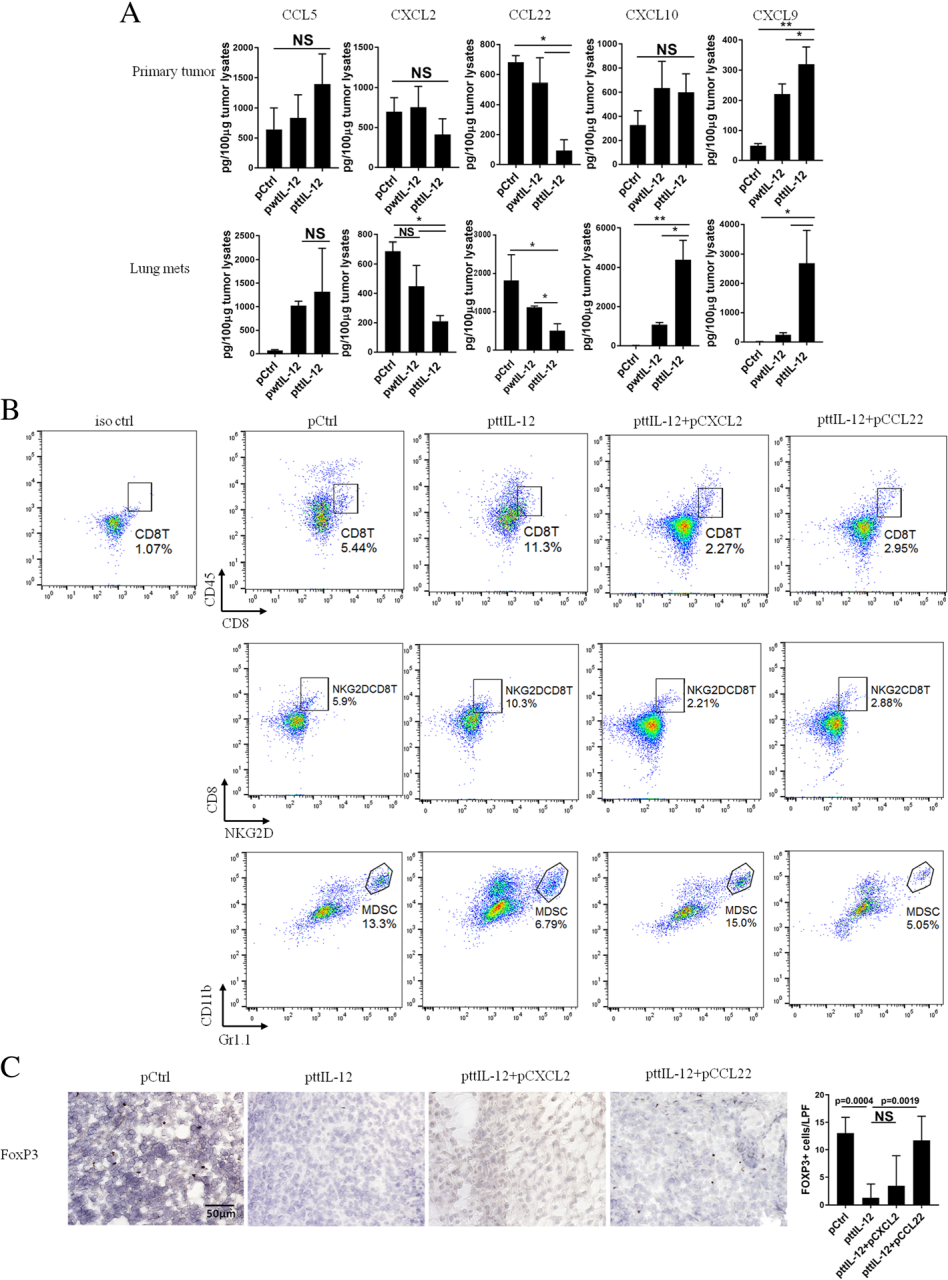
ttIL-12 led to tumor conversion to the CD8^Hi^IFNy^Hi^CD33^Low^FOXP3^Low^ immune profile through enriching CXCL9 and decreasing CXCL2 and CCL22 in tumors. 4 T1 tumor–bearing mice were treated with control pDNA (pCtrl), wild-type IL-12 pDNA (pwtIL-12), tumor-targeted IL-12 pDNA (pttIL-12), or ttIL-12 plus CXCL2 pDNA (pttIL-12 + pCSCL2) or CCL22 pDNA (pttIL-12 + pCCL22). Seven days after the second injection of CXCL2 or CCL22 plasmid DNA, primary tumors and lungs were surgically removed. **a** Levels of CCL5, CXCL2, CXCL9, CXCL10, and CCL22 proteins were determined by ELISA in the primary tumors (upper panels) and lung metastatic nodules (lower panels). **b** Samples of tumors were subjected to flow cytometry to determine percentages of CD8^+^ T cells, NKG2D^+^CD8^+^ T cells (non-exhausted), and myeloid-derived suppressor cells (MDSCs). Proportions of CD8^+^ T cells, NKG2D^+^CD8^+^ T cells, and MDSCs in primary tumors were calculated from flow cytometry data. **c** Primary tumor sections were stained with FOXP3 and numbers of FOXP3^+^ (T regulatory) cells determined by microscopy. Means ± standard error of the mean are shown. NS, not significant; *P < 0.05, **P < 0.01

To understand the mechanism underlying the impact of ttIL-12 treatment on immune profile change in tumors, we used RT-PCR to analyze the tumors for several chemokines and cytokines known to participate in the migration and recruitment of CD8^+^ T cells, Tregs, and MDSCs. Although both ttIL-12 and wtIL-12 significantly increased levels of CCL5, which recruits CD8^+^ T cells into tumors, ttIL-12 treatment was more effective than wtIL-12 treatment in increasing tumor levels of IFNγ and decreasing tumor levels of CCL22 and CXCL2 (Additional file 1: Figure S6B), chemokines that recruit Tregs and MDSCs, respectively. To validate these mRNA-based gene expression results, an ELISA assay was performed. This assay confirmed that ttIL-12 treatment significantly increased the quantities of IFNγ protein (Fig. 2e) and reduced the quantities of CCL22 and CXCL2 proteins in tumors, compared with wtIL-12 treatment (Fig. 5a, Additional file 1: Figure S6C). Moreover, measurement of levels of CXCL9 and CXCL10 proteins, which are CD8^+^ T cell attractants, showed that ttIL-12 increased CXCL9 levels in both primary tumor and lung metastatic nodules in the 4 T1 and LM8 models, while ttIL-12 increased CXCL10 levels only in lung metastatic tumors in the 4 T1 model and liver metastatic tumors in the LM8 model (Fig. 5a, Additional file 1: Figure S6C). Together, these findings indicate that ttIL-12 increases CD8^+^ T cell frequency in tumors and is associated with increases in expression of IFNγ and CXCL9 and that ttIL-12 decreases frequencies of Tregs and MDSCs and is associated with reductions in expression of CCL22 and CXCL2 in the tumor microenvironment.

To further verify the impact of these ttIL-12–induced chemokines on recruitment of MDSCs and Tregs into tumors, pCXCL2 and pCCL22 were administered via intramuscular electroporation in the presence of ttIL-12 therapy to 4 T1 and LM8 tumor–bearing mice. As expected, induced systemic expression of CXCL2 and CCL22 not only decreased frequencies of CD8^+^ T cells, most of which were NKG2D^+^CD8^+^ T cells, but also increased frequencies of MDSCs and Tregs in tumors, respectively, and partially reversed the impact of ttIL-12 (Fig. 5 and Additional file 1: Figure S7). These results suggest that ttIL-12 converts tumors with a short OS immune profile to the long OS immune profile at least partially through reducing CCL22 and CXCL2 expression.

### ttIL-12 treatment coupled with surgery prolongs overall survival

**Fig. 6:**
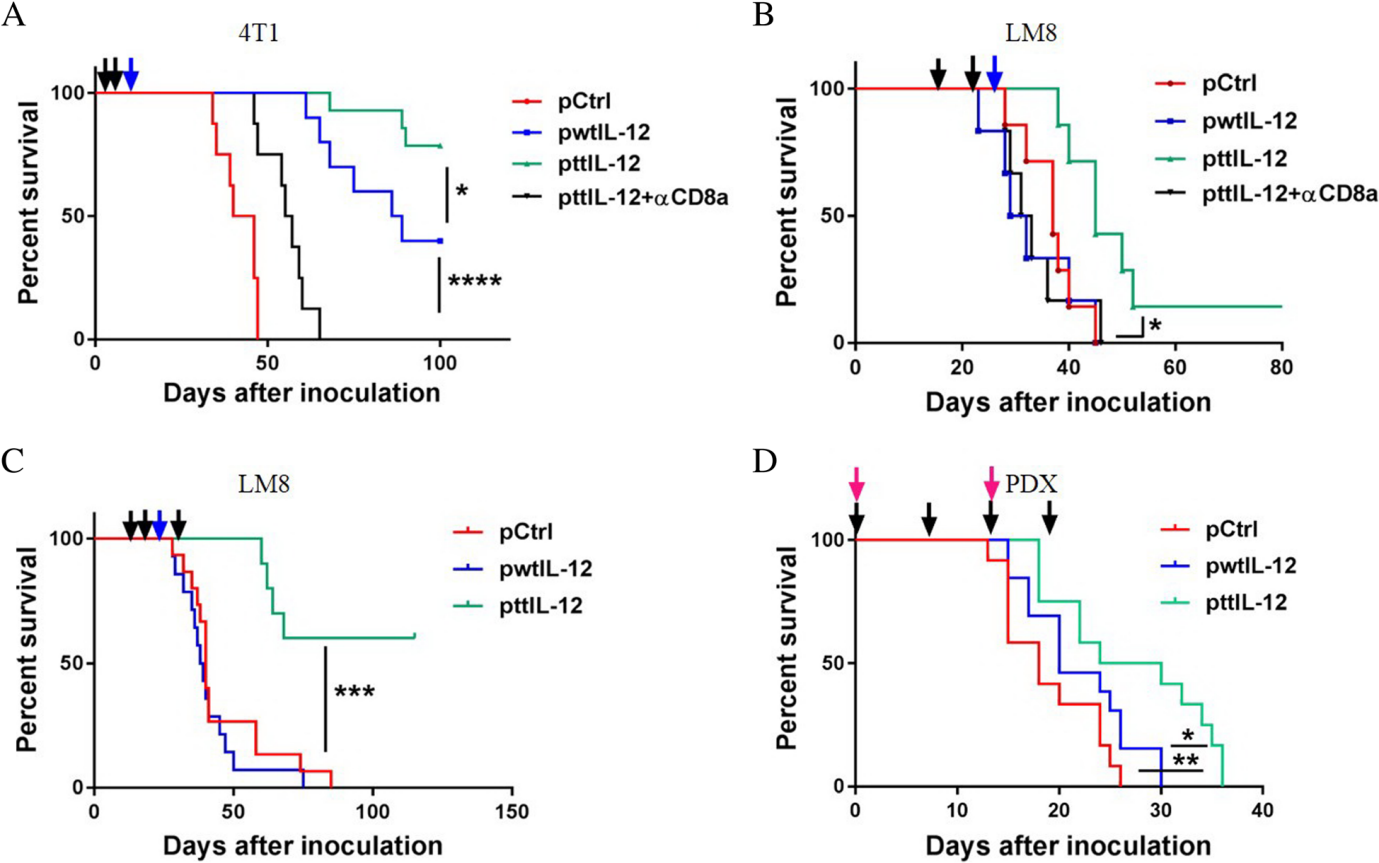
ttIL-12 prolonged survival compared to wtIL-12 in two independent mouse tumor models and a human tumor model. Overall survival of tumor-bearing mice treated with empty plasmid DNA (pDNA; pCtrl), wild-type IL-12 pDNA (pwtIL-12), tumor-targeted IL-12 pDNA (pttIL-12), or pttIL-12 plus a CD8-depleting antibody (pttIL-12 + αCD8a) was determined by using the Kaplan-Meier analysis. Treatment strategy was as described in Fig. 2a. **a** Overall survival of 4 T1 tumor–bearing mice by treatment group. **b** Overall survival of LM8 tumor–bearing mice. **c** Overall survival of LM8 tumor–bearing mice treated with an extra administration of pDNA 15 days after primary tumor removal. (**D**) Overall survival of OS60-SJ osteosarcoma patient-derived xenograft (PDX) tumor-bearing mice overall survival. The dark arrows represent pDNA treatment, the blue arrows represent primary tumor removal, and the red arrows represent PBMC injections. Data represent cumulative results from at least two independent experiments. *P < 0.05, **P < 0.01***P < 0.001, ****P < 0.0001

To verify that inhibition of metastasis and induction of the CD8a^Hi^IFNγ^Hi^CD33^Low^FOXP3^Low^ immune profile by ttIL-12 treatment prolongs OS, we monitored the survival of LM8 and 4 T1 tumor–bearing mice treated with ttIL-12, wtIL-12, or Ctrl in combination with surgery. While both wtIL-12 + S and ttIL-12 + S treatments prolonged survival in the 4 T1 model compared with Ctrl+S treatment (*P* < 0.0001; *n* = 8~14), ttIL-12 + S treatment led to significantly longer OS than wtIL-12 + S treatment (Fig. 6a; *P* = 0.0312). In the LM8 model, mice treated with wtIL-12 + S had curtailed survival compared to those treated with Ctrl+S; this is attributed at least partly to toxicity, because the host mouse strain C3H is very sensitive to IL-12 treatment. However, the C3H mice treated with ttIL-12 + S showed no evidence of toxicity. The reduced toxicity of ttIL-12 treatment may have been due to lower levels of IL-12 in the serum than in mice treated with wtIL-12 (Additional file 1: Figure S6A). Moreover, ttIL-12 + S significantly extended the survival of mice compared with both Ctrl+S and wtIL-12 + S in these tumor-bearing IL-12–sensitive mice (Fig. 6b) (*P* = 0.0086 and *P* = 0.0125, respectively; n = 8~9). To further prolong survival, some LM8 tumor–bearing mice were given ttIL-12 treatment both before and after surgical removal of the primary tumor instead of before surgery only. The extra ttIL-12 treatment after surgery led to a long-term OS rate of 60% (Fig. 6c), showing that ttIL-12 treatment both before and after surgery is more beneficial than ttIL-12 treatment before surgery only.

Furthermore, as shown in Fig. 6d, ttIL-12 significantly prolonged survival of OS60-SJ osteosarcoma tumor–bearing mice compared with either Ctrl treatment or wtIL-12 treatment (*P* = 0.0047 and *P* = 0.0295, respectively; *n* = 12~13), which is in line with the results in the other mouse tumor models. Taken together, these results show that ttIL-12 exhibited high potency in transforming the immune profiles in tumors from IFNγ^Low^CD8^Low^FOXP3^Hi^CD33^Hi^ to IFNγ^Hi^CD8^Hi^FOXP3^Low^CD33^Low^ and suppressing tumor progress in two mouse tumor models and a human tumor model, indicating that ttIL-12 treatment may have the capacity in prolong the survival of patients with cancer when combined with standard-of-care therapy.

## Discussion

In this study, we discovered that the IFNγ^Hi^CD8^Hi^FOXP3^Low^CD33^Low^ immune profile in tumors serves as an excellent prognostic marker for OS across human tumor types. In both mouse and human tumor models, ttIL-12 treatment showed higher potency than wtIL-12 in extending survival of tumor-bearing mice and transforming the immune profile from one associated with short OS to the immune profile associated with long OS in these mice. When combined with surgery, ttIL-12 suppressed tumor progression and metastasis in mouse orthotopic tumor models.

Because cancer, particularly solid tumors, is a heterogeneous disease involving disparate aberrant mutations, such as KRAS and BRAF mutations [32], predicting clinical outcome from a few mutations is difficult. It is now apodictic that cancer is also diverse by nature of its microenvironmental composition, especially immune cell population and activation status [33]. Most therapeutic strategies against tumors have focused on targeting tumor cells directly [34]; however, immune cells in tumors are genetically stable compared with tumor cells and are probably less susceptible to classic mechanisms of treatment resistance. Given the overwhelming heterogeneity of cancer cells, targeting immunosuppressive markers becomes a compelling therapeutic option.

Immunomodulatory therapy has been shown to be an effective option; antibodies or antagonists that block immune checkpoints, such as the cytotoxic T lymphocyte-associated antigen 4 (CTLA4) inhibitor ipilimumab [35], programmed death 1 receptor (PD-1) inhibitor nivolumab [36, 37], and programmed death 1 ligand 1 (PD-L1) inhibitor lambrolizumab [38, 39], or chemokine or chemokine receptors inhibitors, such as the CXC-chemokine receptor 4 (CXCR4) antagonist LY2510924 [40] and CXCL8 blocker repertaxin [41], have been approved by the U.S. Food and Drug Administration or are being investigated in clinical trials [33, 42]. However, the rate of response to immune checkpoint blockers is low in most tumor types [43]. The mechanism underlying the low response rate is not clear, but it seems that T cell infiltration into tumors affects the clinical outcome of PD-L1 and PD-1 blockade therapy. The question is how to increase the numbers of CD8^+^ T cells in tumors to overcome this resistance. One of the simplest approaches is to simply administer exogenous T cells, such as CAR-T cells, but the clinical impact of such treatment against solid tumors is minimal [44]. The poor results may be due to multiple reasons, but one major reason is the failure of the administered T cells to infiltrate into tumors [45]. Therefore, the therapeutic impact of several chemokines that recruit CD8^+^ T cell into tumors have been explored [46]. As we have found here, CXCL9 and CXCL10 may be important for CD8^+^ T cell recruitment (Fig. 5).

High numbers of CD8^+^ tumor-infiltrating lymphocytes is often an indicator of good response to various therapies, but not always [47]. Two studies found that a high number of CD8^+^ T cells was barely associated with clinical outcome [48], which indicates that the presence of CD8^+^ T cells in tumors will not guarantee tumor eradication and long-term survival. Since poor outcome may be due to CD8^+^ T cell exhaustion or inactivation, both markers of CD8^+^ T cell activation/exhaustion and of suppressive immune cells should be included in the optimal prognostic immune profile. Scott et al. found that a high immune gene cluster expression summary score can predict OS [3]. However, second-generation gene sequencing is needed to get the score, which makes it costly and laborious for the prediction of clinical outcome. We found here, through comparing different immune profiles, that the IFNγ^Hi^CD8^Hi^FOXP3^Low^CD33^Low^ immune profile in tumors was a compelling predictor of survival across tumor types. Thus, reprogramming or re-educating tumors to the IFNγ^Hi^CD8^Hi^FOXP3^Low^CD33^Low^ immune profile may represent an exciting new opportunity in tumor immunotherapy.

Although this study found that treatment with ttIL-12, but not wtIL-12, can effectively convert both primary and metastatic tumors to this long-term survival–associated immune profile (Fig. 6), this treatment alone will not eliminate large primary tumors. Thus, standard-of-care surgical removal of primary tumors is still needed. We propose that transformation of the immune profile with ttIL-12 may boost the efficacy of other standard-of-care therapeutic modalities such as chemotherapy, targeted therapy, and radiation; however, this suggestion must be evaluated experimentally. Though a number of IL-12–based therapies, such as IL-12 gene therapy, IL-12–transduced autologous fibroblasts, adenovirus encoding IL-12, and a combination of IL-12 and peptide vaccine, are now being evaluated in clinical trials, only a few have shown encouraging results [49]. This may be because the therapeutic dose of IL-12 may induce toxicity due to high IL-12 levels in serum. Thus, reduction of IL-12 levels in serum and induction of IL-12 expression in tumors is a big challenge in overcoming the drawbacks of IL-12 treatment. In our study, ttIL-12 treatment, compared with wtIL-12, induced higher IL-12 levels at the tumor sites and lower IL-12 levels in serum (Fig. 2e, Additional file 1: Figure S6A), which was accomplished by the tumor-anchoring effect of the CSV-targeting carcinoma homing peptide expressed by ttIL-12. Offering enhancement of the wtIL-12 antitumor effect and reduction of toxicity, ttIL-12 has promise as a new form of anticancer immunotherapy.

## Conclusions

Tumor CSV-targeted IL-12, when combined with surgery, led to conversion of tumors to the IFNγ^Hi^CD8^Hi^FOXP3^Low^CD33^Low^ immune profile, enhancement of the capacity to eliminate relapse/metastasis, and decreased systemic toxicity in both mouse and human tumor models.

## Additional file

Additional file 1: **Table S1.** Validation of CD8^Hi^IFNy^Hi^CD33^Low^FOXP3^Low^ immune profile in TCGA data sets. **Figure S1.** ttIL-12 enhanced suppression of primary tumor growth. **Figure S2.** ttIL-12 decreased M2 macrophages in 4 T1 tumors. **Figure S3.** ttIL-12 enhanced CD8+ T cell infiltration, decreased MDSCs, M2 macrophages infiltration in primary LM8 tumors. **Figure S4.** ttIL-12 increased activating CD8 T cells and decreased Tregs in lung metastatic nodules. **Figure S5.** ttIL-12 increased NKG2D + CD8+ T cell infiltration and decreased MDSC and Tregs infiltration into liver metastatic nodules. **Figure S6.** ttIL-12 led enriched CXCL9 and decreased CXCL2 and CCL22 in both primary tumors and metastatic tumors. **Figure S7.** Overexpression of CXCL2 and CCL22 reversed ttIL-12’s efficacy on increasing CD8 T cells and decreasing MDSCs and Tregs in LM8 tumors. **Figure S8.** ttIL-12 increased IFNγ level, enhanced CD8+ T cell infiltration, and decreased MDSCs and Tregs infiltration in osteosarcoma PDX tumors. (DOCX 15277 kb)

## Data Availability

Not applicable.

## Abbreviations

Arg1: Arginase 1
CCL22: CC-chemokine ligand 22
CCL5: CC-chemokine ligand 5
CSV: Cell-surface vimentin
CTLA4: Cytotoxic T lymphocyte-associated antigen 4
Ctrl: Control pDNA
CXCL10: CXC-chemokine ligand 10
CXCL2: CXC-chemokine ligand 2
CXCL9: CXC-chemokine ligand 9
CXCR4: CXC-chemokine receptor 4
httIL-12: Human ttIL-12
hwtIL-12: Human wtIL-12
IFNγ: Interferon-gamma
IL-10: Interleukin-10
IL-12: Interleukin-12
IL-35: Interleukin-35
MDSCs: Myeloid-derived suppressor cells
NK: Natural killer
NKG2D: Natural killer group 2D
OS: Overall survival
PBMCs: Peripheral blood mononuclear cells
pCCL22: CCL22 plasmid DNA
pCXCL2: CXCL2 plasmid DNA
PD-1: Programmed death 1 receptor
PD-L1: PD-1 ligand
pDNA: Plasmid DNA
PDX: Patient-derived xenograft
SD: Standard deviation
TCGA: The Cancer Genome Atlas
TGF-β: Transforming growth factor beta
Tregs: Regulatory T cells
ttIL-12 + S: ttIL-12 treatment combined with surgery
ttIL-12: Tumor cell surface vimentin–targeted interleukin 12
wtIL-12 + S: wtIL-12 treatment combined with surgery
wtIL-12: Wild-type interleukin 12
