# Supplementary material for: Tumor-targeted IL-12 combined with tumor resection yields a survival-favorable immune profile
Source: J Immunother Cancer. 2019 Jun 17;7:154. doi: 10.1186/s40425-019-0631-z (PMC6580640; doi:10.1186/s40425-019-0631-z)
Supplement: Supplementary file 1 — Table S1. Validation of CD8HiIFNyHiCD33LowFOXP3Low immune profile in TCGA data sets. Figure S1. ttIL-12 enhanced suppression of primary tumor growth. Figure S2. ttIL-12 decreased M2 macrophages in 4 T1 tumors. Figure S3. ttIL-12 enhanced CD8+ T cell infiltration, decreased MDSCs, M2 macrophages infiltration in primary LM8 tumors. Figure S4. ttIL-12 increased activating CD8 T cells and decreased Tregs in lung metastatic nodules. Figure S5. ttIL-12 increased NKG2D + CD8+ T cell infiltration and decreased MDSC and Tregs infiltration into liver metastatic nodules. Figure S6. ttIL-12 led enriched CXCL9 and decreased CXCL2 and CCL22 in both primary tumors and metastatic tumors. Figure S7. Overexpression of CXCL2 and CCL22 reversed ttIL-12’s efficacy on increasing CD8 T cells and decreasing MDSCs and Tregs in LM8 tumors. Figure S8. ttIL-12 increased IFNγ level, enhanced CD8+ T cell infiltration, and decreased MDSCs and Tregs infiltration in osteosarcoma PDX tumors. (DOCX 15277 kb) [file 40425_2019_631_MOESM1_ESM.docx]

**Supplementary Materials**

**Tumor models:**

Orthotopic 4T1and LM8 tumors were initiated by inoculating 1×10^5^ cells in the 3^rd^ mammary fat pads of the BALB/c mice and in the right tibia of C3H mice, respectively. When 4T1 tumors were 3–4 mm in diameter (day 5 after tumor cell inoculation), and when LM8 tumors in the tibia were detected by X-ray (day 10 after tumor cell inoculation), the mice received the first of pDNA treatments (10 μg; wtIL-12, ttIL-12, or Ctrl) via intramuscular electroporation as described previously; a second identical treatment was administered 10 days later. Tumor volumes were determined twice per week. Seven days after the second treatment, the primary tumors were removed surgically and the wounds sealed with wound clips. The removed primary tumors were used for later analysis. Survival duration of mice was monitored twice per week. For metastatic analysis, 4T1-bearing mice and LM8-bearing mice were euthanized twenty days and five days after primary tumor removal respectively, and lungs, livers and bones were collected to analyze metastatic status. India ink inflation was performed to determine the level of lung metastasis, and white metastatic nodules were counted using a dissecting microscope.

For PDX model, patient derived OS60-SJ osteosarcoma tumor line were generously provided by the [Pediatric Preclinical Testing Consortium](http://www.google.com/url?sa=t&rct=j&q=&esrc=s&source=web&cd=4&ved=2ahUKEwj50Lmw_9DcAhURRqwKHXVyAGUQFjADegQICRAB&url=http%3A%2F%2Fwww.ncipptc.org%2F&usg=AOvVaw02k-X3NfAzy9y-buhLU_Ec) (Dr. Richard Gorlick, MD Anderson Cancer Center). This tumor line is maintained by serial passage in severe combined immune deficient (SCID) mice. CB17SC *scid^−/−^* female mice were used to propagate subcutaneously implanted OS60-SJ osteosarcoma tumors. Briefly, sterile scalpel was used to cut the tumor into pieces with the size of 8mm^3^. Insert one individual piece of tumor into the subcutaneous layer on the backs of CB17SC *scid^−/−^* female mice. When tumor sizes reached 300mm^3^, Ctrl, hwt-IL12, httIL-12 DNA plasmid treatment was performed as described above once per week for 4 weeks. To make these CB17SC *scid^−/−^* mice as immunocompetent mice, 2×10^7^ human PBMCs were injected into the CB17SC *scid^−/−^* mice intra vein every 2 weeks for twice along with pDNA treatment.

**The primer sequences of all mouse genes:**

mCXCL2: F: AACATCCAGAGCTTGAGTGTGA, R: TTCAGGGTCAAGGCAAACTT

mCXCL9: F: AGCAGTGTGGAGTTCGAGGAA, R: GGTGCTGATGCAGGAGCAT

mCXCL10: F: GACGGTCCGCTGCAACTG, R: CCCTATGGCCCTCATTCTCA

mCCL22: F: CTCTGCCATCACGTTTAGTGAA, R: GACGGTTATCAAAACAACGCC

mIFNγ: F: CTGCTGATGGGAGGAGATGTCT, R: TGCTGTCTGGCCTGCTGTTA

mTGFβ1: F: CGGAGAGCCCTGGATACCA, R: GCCGCACACAGCAGTTCTT

mGAPDH: F: CCAGCCTCGTCCCGTAGAC, R: CGCCCAATACGGCCAAA

**Supplementary table 1. Validation of CD8^Hi^IFNy^Hi^CD33^Low^FOXP3^Low^ immune profile in TCGA data sets**

**

**

**Supplementary figure S1.**

**
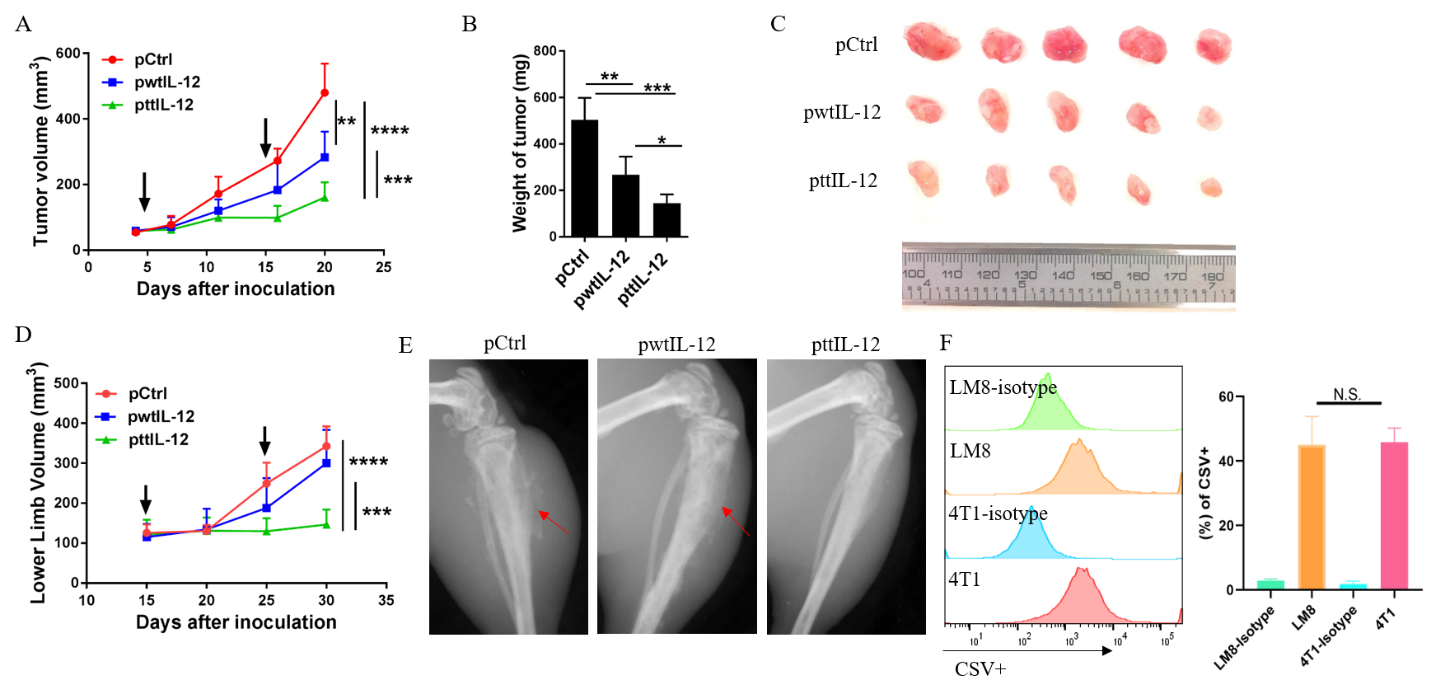
**

**Supplementary figure S1. ttIL-12 enhanced suppression of primary tumor growth.** (A) Tumor growth monitored over time showed an enhanced reduction in size of 4T1 inoculations in mice treated with pttIL-12 compared to pwtIL-12. Data are shown as average volumes (symbols, n = 5) ± SD. (B) Tumor weights of 4T1 inoculations (n = 5) are shown as averages ± SD. (C) Tumors of 4T1 inoculations resected 22 days after inoculation. (D) Tumor growth monitored according to the limb volume of LM8 inoculations. Data are shown as average volumes (symbols, n = 5~8) ± SD. (E) Tumors of LM8 inoculations were monitored using X-Ray. *P <0.05, **P <0.01, ***P <0.001, ****P <0.0001. (F) Representative flow cytometry plots of CSV level of LM8 cells and 4T1 cells are shown in left; quantitative analysis data is shown in right (n=3).

**Supplementary figure S2.
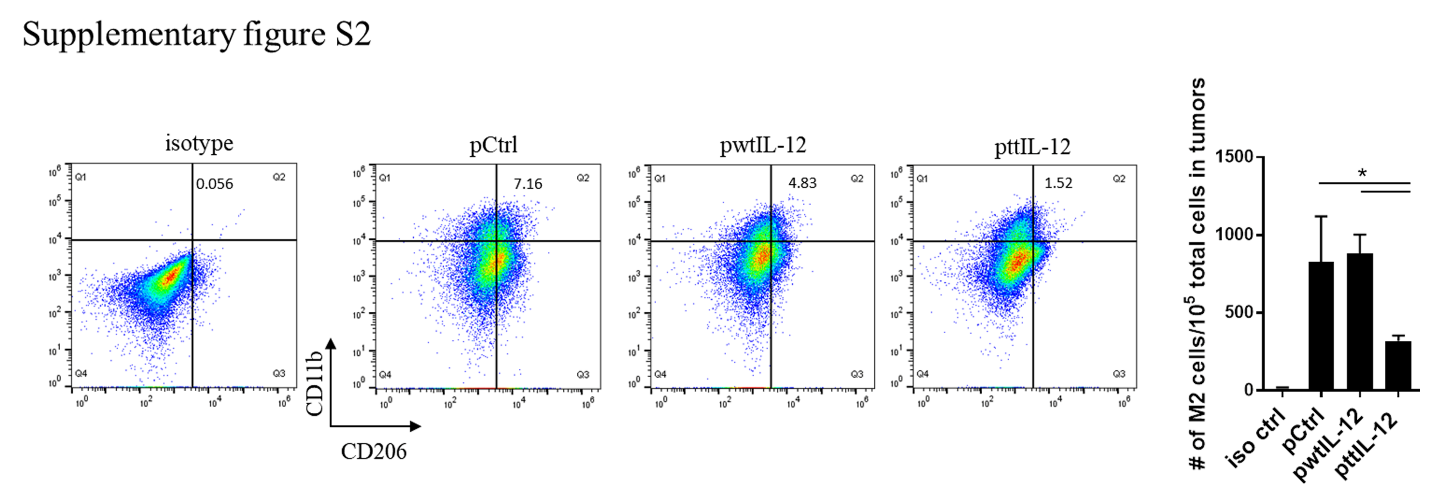
**

**Supplementary figure S2. ttIL-12 decreased M2 macrophages in 4T1 tumors.** Representative flow cytometry plots of CD11b+CD206+ (M2 macrophages) proportion in 4T1 orthotopic tumors; absolute numbers of M2 macrophages per 10^5^ total tumor cells were calculated from the flow cytometry data (n=3). *P <0.05.

**Supplementary figure S3.**


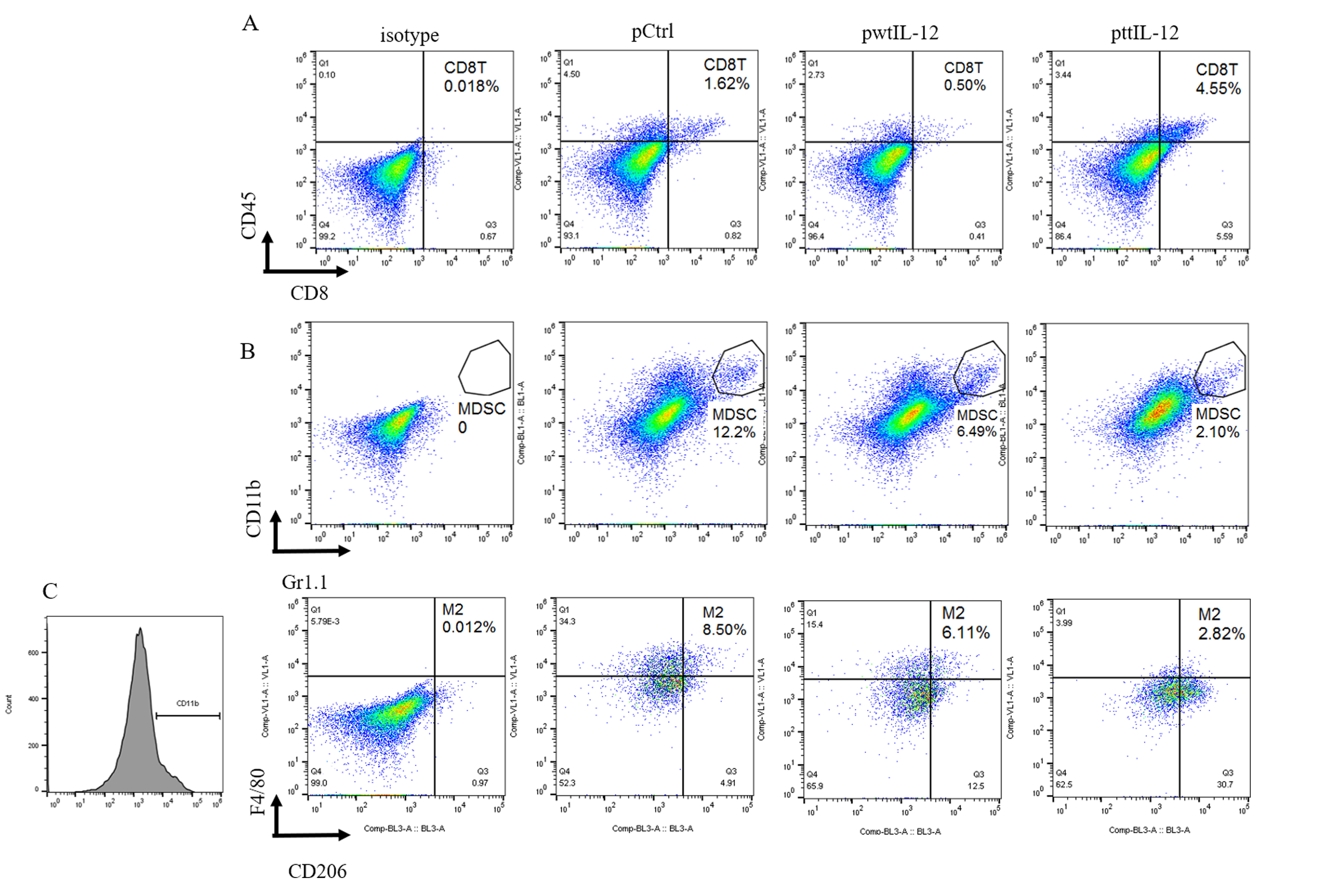


**Supplementary figure S3. ttIL-12 enhanced CD8+ T cell infiltration, decreased MDSCs, M2 macrophages infiltration in primary LM8 tumors.** Representative flow cytometry plots of percentages of CD8 T cells (A), MDSCs (B) and M2 macrophages (C) in LM8 orthotopic tumors.

**Supplementary figure S4
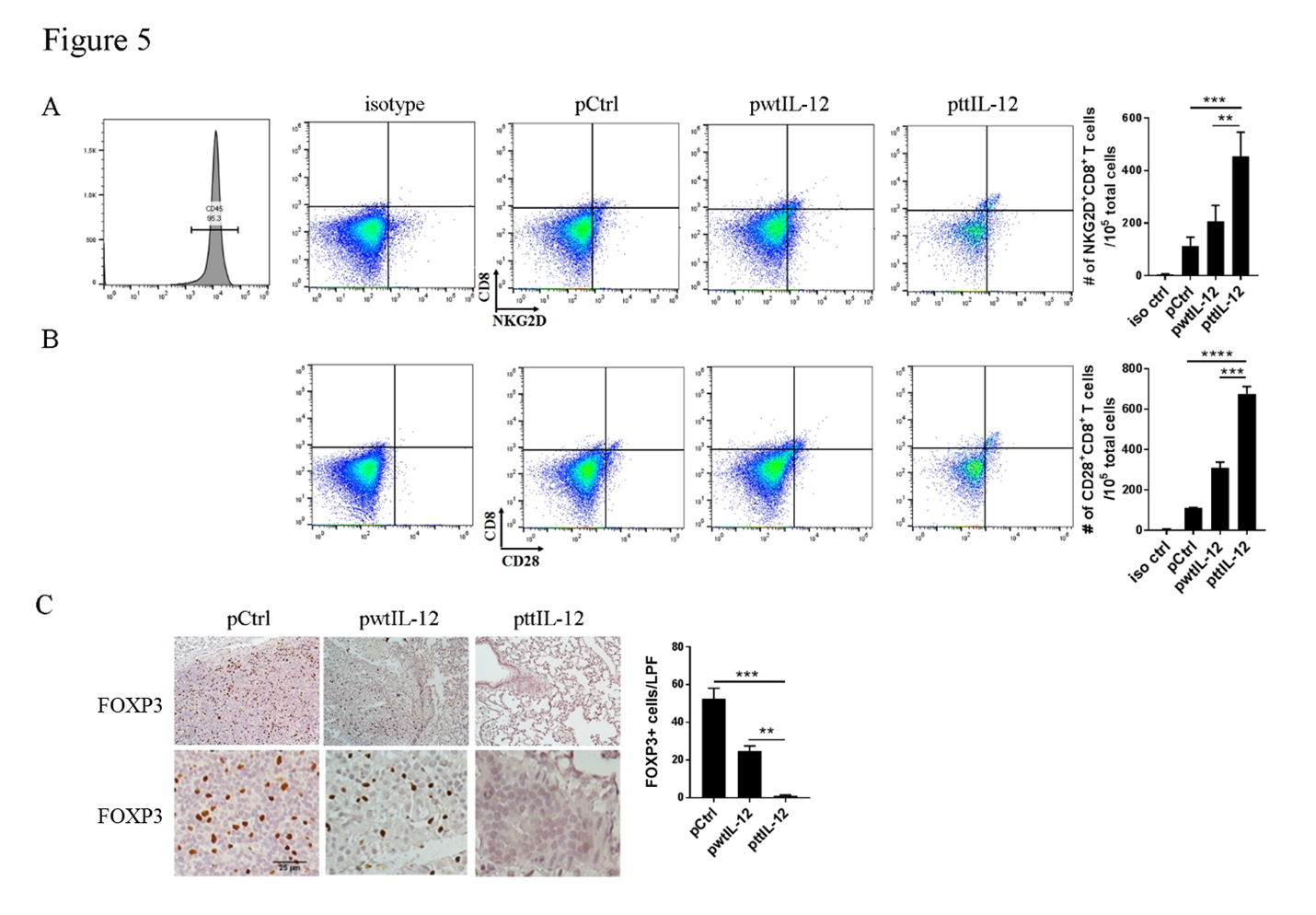
**

**Supplementary figure S4. ttIL-12 increased activating CD8 T cells and decreased Tregs in lung metastatic nodules.** (A, B) Lung metastatic nodules from treated 4T1 tumor-bearing mice were subjected to flow cytometry to determine percentages of CD8+CD28+ and CD8+NKG2D+ cells, and the absolute numbers of the indicated cell subsets were calculated from the flow cytometry data. (C) Sections of paraffin-embedded lung from 4T1 tumor-bearing mice were labeled with FoxP3, and absolute numbers of FoxP3+ cells in metastases were determined by microscopy. Representative images are shown. Scale bars, 100 μm. Pooled data from two independent experiments with n = 3 mice per treatment group. Means ± SEM are shown. **P <0.01, ***P <0.001, ****P <0.0001.

**Supplementary figure S5.
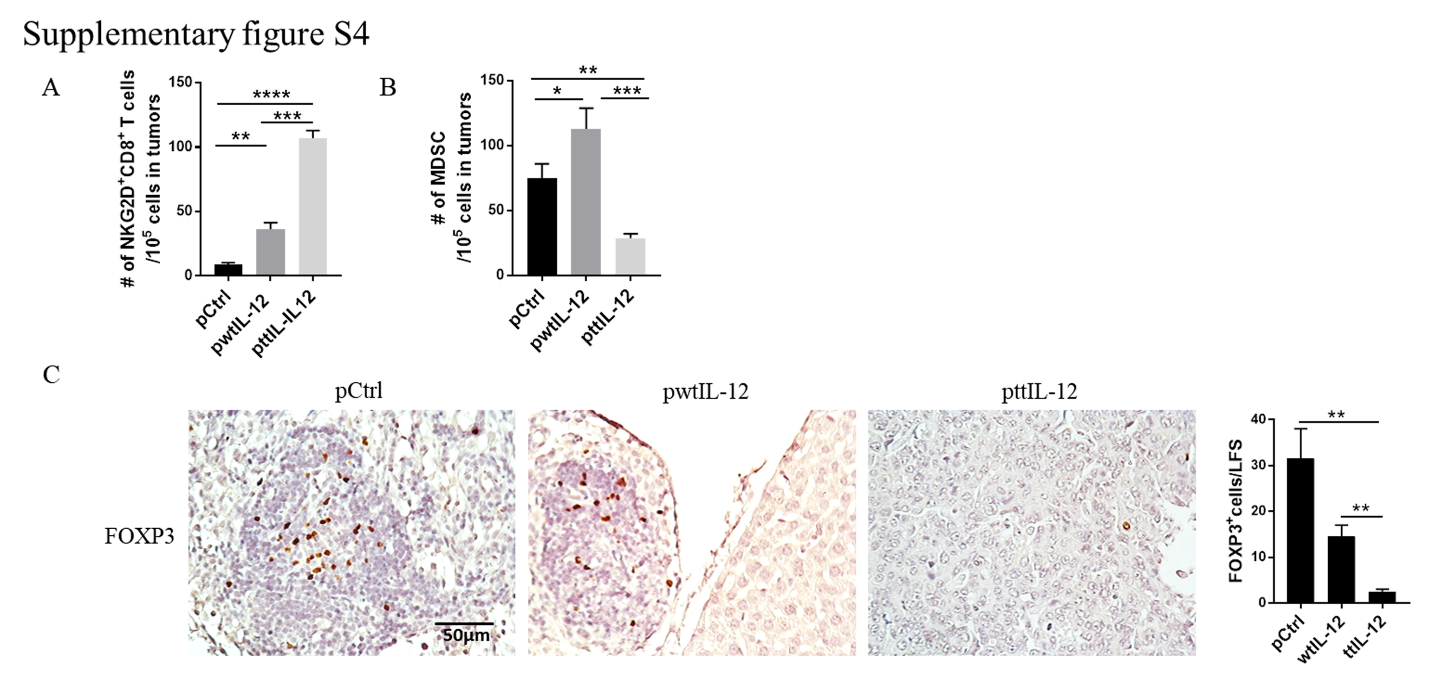
**

**Supplementary figure S5. ttIL-12 increased NKG2D+CD8+ T cell infiltration and decreased MDSC and Tregs infiltration into liver metastatic nodules.** (A, B) The livers of mice bearing LM8 tumors were subjected to flow cytometry to determine percentages of NKG2D+CD8+ T cells and CD11b+Gr1+ (MDSCs) in metastatic nodules, and absolute numbers of NKG2D+CD8+ T cells and MDSCs were calculated from the flow cytometry data. (C) Sections of paraffin-embedded livers from LM8 tumor-bearing mice were labeled with FoxP3, and absolute numbers of FoxP3+ cells in metastases were determined by microscopy. Representative images are shown. Scale bars, 50μm. Means ± SEM are shown. **P < 0.01.

**Supplementary figure S6.
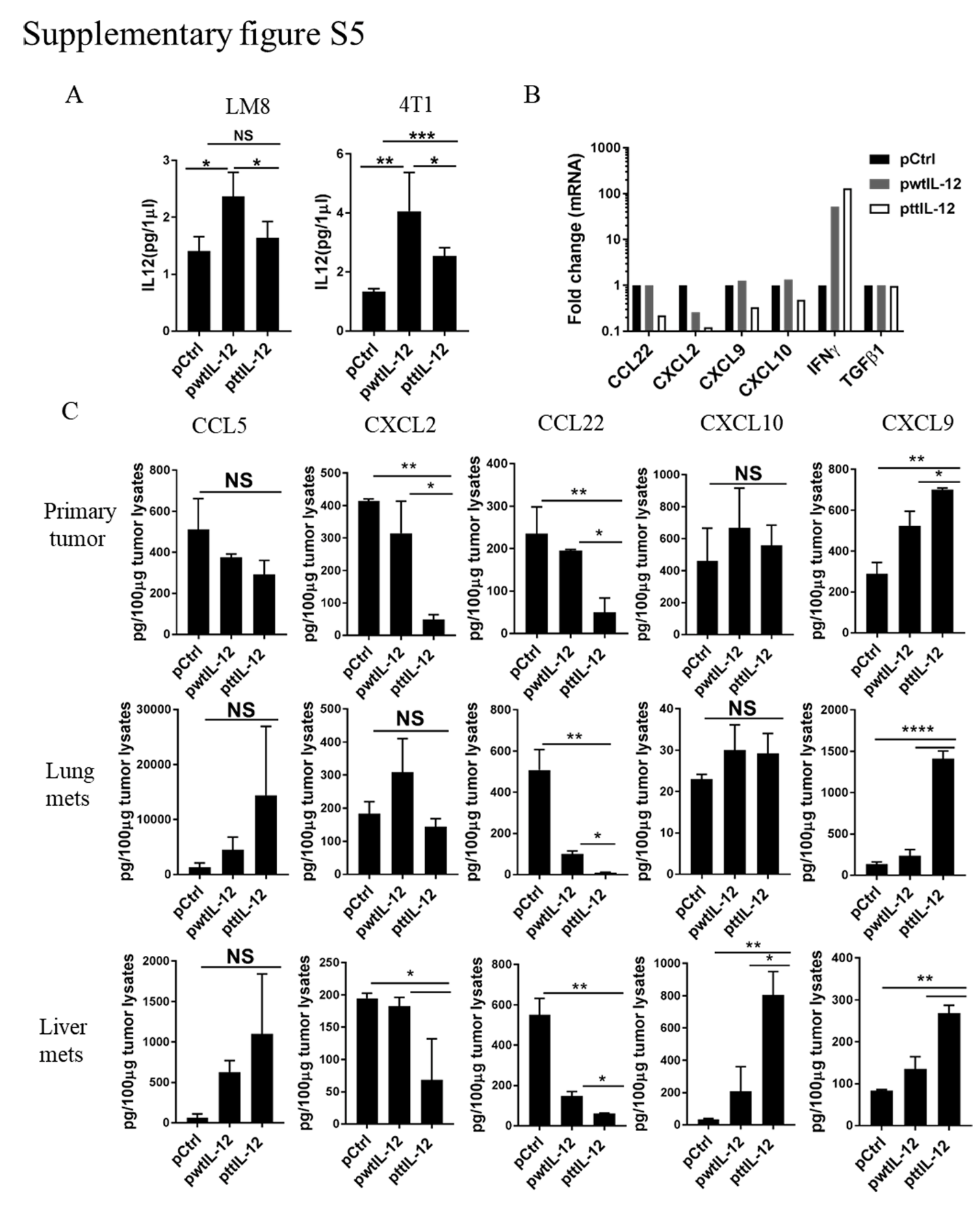
**

**Supplementary figure S6. ttIL-12 led enriched CXCL9 and decreased CXCL2 and CCL22 in both primary tumors and metastatic tumors.** (A) Levels of the IL-12 proteins in serum of LM8-bearing mice and 4T1-bearing mice were measured by ELISA. (B) mRNA levels of CCL22, CXCL2, CXCL9, CXCL10, IFNγ and TGFβ1, CCL5 in 4T1 primary tumors were detected using real-time PCR. (C) Levels CCL5, CXCL2, CXCL9, CXCL10, and CCL22 proteins in the primary tumors (upper panels), lung metastatic nodules (middle panels) and liver metastatic nodules (bottom panels) of LM8 tumor-bearing mice were determined by ELISA. *P <0.05, **P <0.01, ***P <0.001, ****P <0.0001.

**Supplementary figure. S7.**

**Supplementary figure. S7. Overexpression of CXCL2 and CCL22 reversed ttIL-12’s efficacy on increasing CD8 T cells and decreasing MDSCs and Tregs in LM8 tumors.** (A)Seven days after the 2^nd^ injection of CXCL2 DNA plasmid and CCL22 DNA plasmid, primary tumors from the LM8 orthotopic model were surgically removed and subjected to flow cytometry to determine percentages of CD8+ T cells and MDSCs. (B) Tregs in the primary tumor sections were detected by microscopy. Scale bars, 50 μm for high magnification IHC. Means ± SEM are shown. **P <0.01, ****P <0.0001.


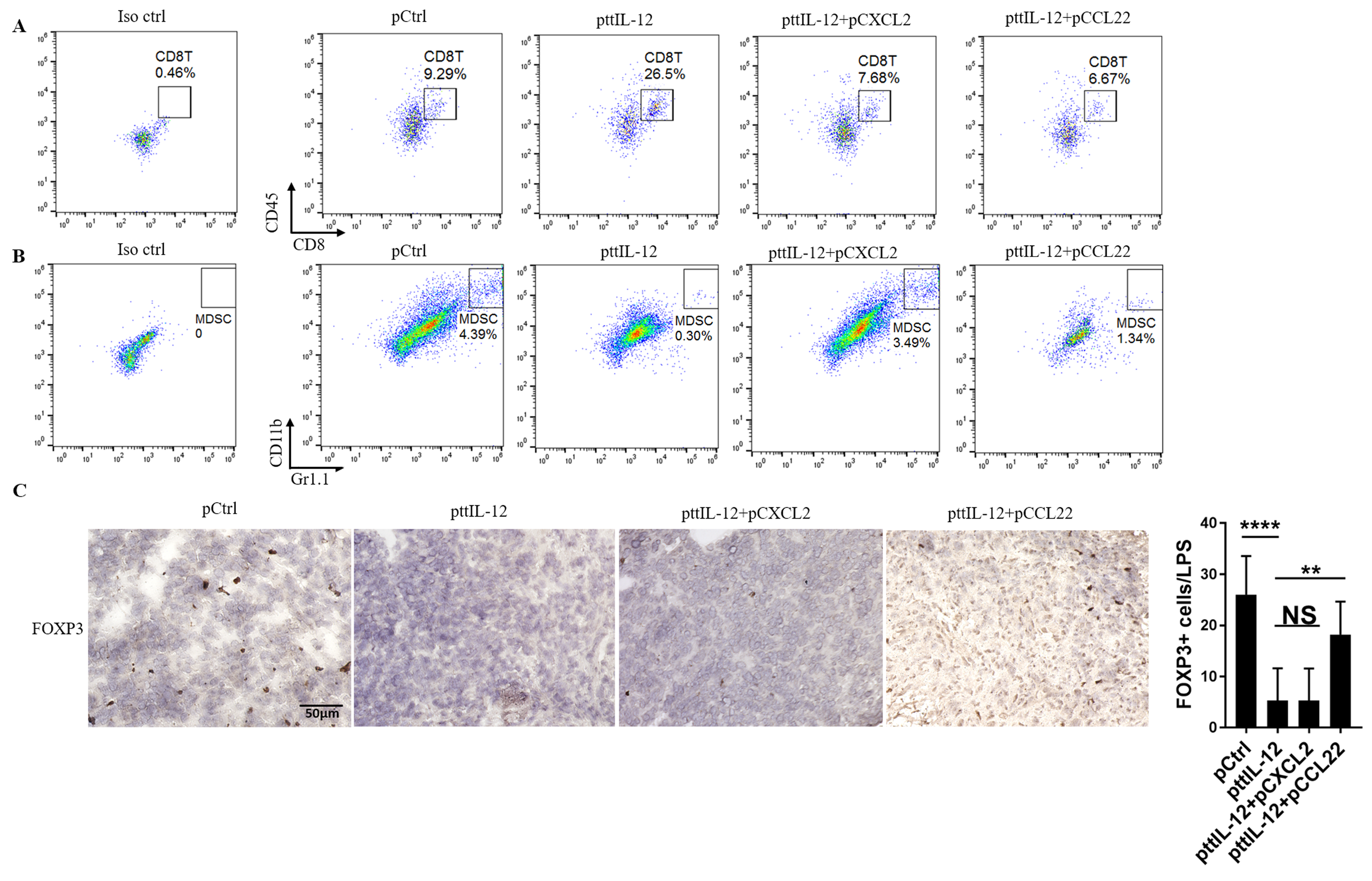


**Supplementary figure S8.**


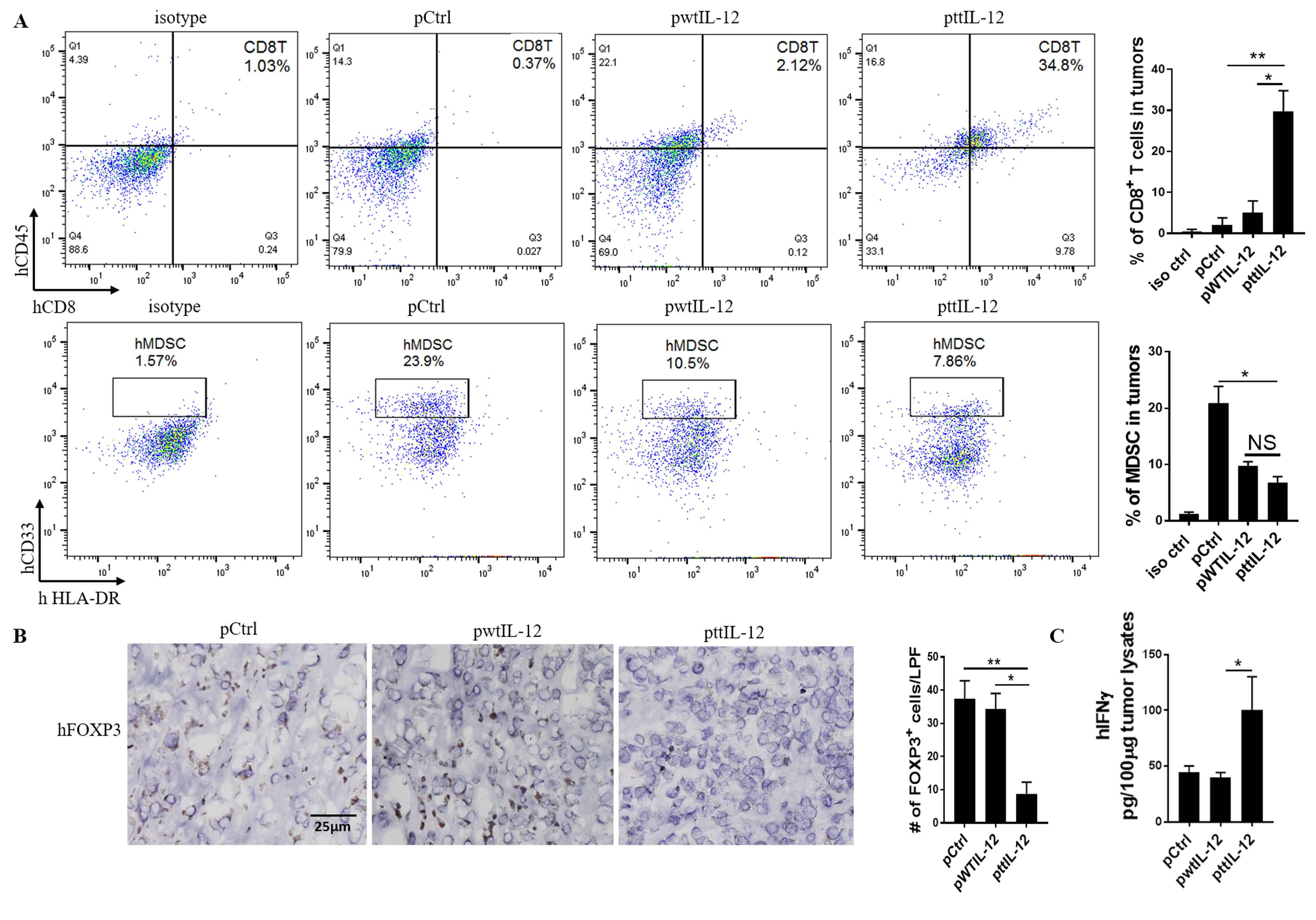


**Supplementary figure S8. ttIL-12 increased IFNγ level, enhanced CD8+ T cell infiltration, and decreased MDSCs and Tregs infiltration in osteosarcoma PDX tumors.** Seven days after the 4^th^ treatment of Ctrl, hwt-IL12, and httIL-12DNA plasmid, tumors from osteosarcoma PDX model were surgically removed and subjected to Elisa, flow cytometry and immunohistology. (A) Representative flow cytometry plots of CD45+CD8+ T cells and CD11b+CD33+HLA- (MDSCs) frequency. (B) FOXP3+ Tregs were detected. (C) IFNγ protein levels were measured by ELISA. Scale bars, 20μm for high magnification IHC. Means ± SEM are shown. *P <0.05, **P <0.01.
